# Genistein Inhibits the Pathogenesis of *Aeromonas hydrophila* by Disrupting Quorum Sensing Mediated Biofilm Formation and Aerolysin Production

**DOI:** 10.3389/fphar.2021.753581

**Published:** 2021-09-28

**Authors:** Jing Dong, Defu Zhang, Jianrong Li, Yongtao Liu, Shun Zhou, Yibin Yang, Ning Xu, Qiuhong Yang, Xiaohui Ai

**Affiliations:** ^1^ Yangtze River Fisheries Research Institute, Chinese Academy of Fishery Sciences, Wuhan, China; ^2^ College of Food Science and Engineering, Bohai University, Jinzhou, China

**Keywords:** *Aeromonas hydrophila*, quorum sensing, genistein, anti-virulence, natural compound

## Abstract

*Aeromonas hydrophila* is an opportunistic pathogen that is responsible for a variety of infectious diseases both in human and animals, particularly aquatic animals. Moreover, the pathogen has become a foodborne pathogen by transmitting from seafood to human. The abuse of antibiotics in aquaculture results in the emergence of antibiotic resistance and treatment failure. Therefore, novel approaches are urgently needed for managing resistant *A. hydrophila* associated infections. Aerolysin, an essential virulence factor of pathogenic *A. hydrophila* strain, has been identified as target developing novel drugs against pathogenesis of *A. hydrophila*. In the present study, genistein, without anti-*A. hydrophila* activity, was identified that could decrease the production of aerolysin and biofilm formation at a dose-dependent manner. Transcription of aerolysin encoding gene *aerA* and quorum sensing related genes *ahyI* and *ahyR* was significantly down-regulated when co-cultured with genistein. Cell viability studies demonstrated that genistein could significantly improve aerolysin mediated A549 cell injury. Furthermore, genistein could provide a remarkable protection to channel catfish infected with *A. hydrophila*. These findings indicate that targeting quorum sensing and virulence can be a useful approach developing drugs against *A. hydrophila* infections in aquaculture. Moreover, genistein can be chosen as a promising candidate in developing drugs against *A. hydrophila*.

## 1 Introduction

Aquatic products have become one of the major sources of high quality proteins for human, playing critical role in global food security, particularly in developing countries. China has become the largest producer and exporter of aquatic products all over the world since 2015 (Mo et al., 2018). However, diseases caused by bacterial pathogens threatened the healthy development of aquaculture industry and safety of aquatic products. Therefore, controlling of bacterial infection in aquaculture can not only reduce the mortality of cultured fish, but decrease the risks of foodborne infection. *Aeromonas hydrophila* (*A. hydrophila*), the leading cause of bacterial infections in freshwater aquaculture, is widely distributed in nature, particular aquatic environments (Velazquez et al., 2001). The bacterium is responsible for a number of diseases in aquatic animals, such as hemorrhagic septicemia, exophthalmia and dropsy (Patel et al., 2017). Moreover, *A. hydrophila* can be transmitted from uncooked aquatic food to human, and has been considered as a foodborne bacteria associated with several human infections (Kuijper et al., 1990; Janda, 1991). Antibiotics are the main approach treating bacterial infections. However, the emergence of antibiotic resistance limits the use of antibiotics and results in treatment failure. Therefore, antibiotic alternative strategies are needed to overcome resistant *A. hydrophila* strains.

Virulence factors are involved in the infectious processes such as colonization, invasion and persistence in the hosts (Fleitas Martinez et al., 2019). Therefore, anti-virulence strategies have been identified as a novel approach in developing drugs against resistant bacterial infections. The pathogenesis of *A. hydrophila* is determined by the amount of virulence factors (Zhao et al., 2020). Among which, aerolysin has been best characterized and is considered as one of the most critical virulence factors for establishing infections (Chakraborty et al., 1987; Fivaz et al., 2001). Aerolysin is secreted as an inactive precursor naming proaerolysin, the activity of the toxin will release after cleaving the 43 residues at the C-terminus of the protein by trypsin or furin (Iacovache et al., 2011). After activation, the toxin can form a homo-heptameric pore on target cells and results in cell death, a number of Mammalian cells are sensitive to the toxin (Wuethrich et al., 2014). Therefore, aerolysin has been identified as an ideal target developing drugs against *A. hydrophila* infections. Moreover, biofilm formation and the expression of aerolysin and several other virulence factors are regulated by quorum sensing (QS) system (Rama Devi et al., 2016). A number of studies have demonstrated that anti-QS compounds are a useful approach to overcome the bacterial resistance problem (Ding et al., 2017; Patel et al., 2017; Ding et al., 2018).

**FIGURE 1:**
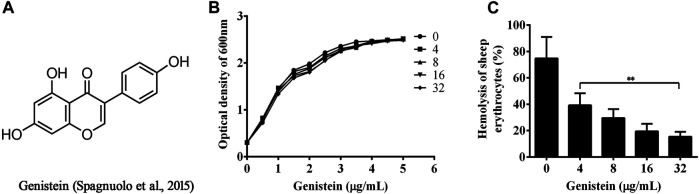
Inhibitory effects of genistein on hemolytic activity of bacterial supernatants co-cultured with genistein. **(A)**, chemical structure of genistein; **(B)**, Growth curves of *A. hydrophila* XS-91-4-1 plus indicated concentrations of genistein; **(C)**, genistein inhibits hemolytic activity of bacterial supernatants co-cultured with genistein, 1% Triton X-100 treated red blood cells represented 100% hemolytic activity, three independent hemolytic assays were performed and data in Figure 1C were mean value ±SD, *p < 0.05 and **p < 0.01 when compared with the genistein-free supernatants.

Herbal medicine and natural compounds have been used as a source of medicine and make a significant contribution to the pharmaceutical industry (Machado et al., 2020). Genistein (Figure 1A), an isoflavone compound, can be isolated from most of leguminous plant foods (Spagnuolo et al., 2015). Soybean has been reported with the highest concentrations of genistein. Previous studies have demonstrated that genistein showed a variety of biological activities, including anti-cancer, anti-inflammation, anti-apoptosis and anti-oxidation activities (Wei et al., 1993; Ruiz-Larrea et al., 1997; Li et al., 2006). However, there is little knowledge of genistein against bacterial pathogens. In the present study, we found that genistein had no role on *A. hydrophila* growth at concentrations lower than 512 μg/ml, could reduce the production of aerolysin and biofilm formation of *A. hydrophila* by inhibiting the QS system. Moreover, genistein could provide a significant protection to A549 cells and fish infected with *A. hydrophila*.

## 2 Materials and Methods

### 2.1 Microorganism and Reagents

*A. hydrophila* strain XS-91-4-1 was kindly provided by Prof. Aihua Li at Institute of Hydrobiology, Chinese Academy of Sciences. Genistein (CAS No. 446-72-0) with purity of 99.5% was purchased from National Institutes for Food and Drug Control (Beijing, China). Enrofloxacin (CAS No. 93106-60-6) was purchased from Sigma-Aldrich (St. Louis, MO, United States). For *in vitro* studies, Genistein and enrofloxacin were prepared as stock solutions at concentrations of 40.96 mg/ml by DMSO. For *in vivo* study, genistein was dissolved in sterile PBS.

### 2.2 Determination of Minimal Inhibitory Concentrations of Enrofloxacin and Genistein

Micro-dilution method was used to assess the MICs of enrofloxacin and genistein against *A. hydrophila* XS-91-4-1 under the guidance of CLSI (2009). In brief, enrofloxacin and genistein were diluted at 2-folder by MHB medium in 96-well plate to reach concentrations ranging from 32 to 0.125 μg/ml and 512 to 2 μg/ml, respectively. Bacterial cells were collected by centrifugation and re-suspended in MHB medium to obtain a density of about 5 × 10^5^ CFU/ml by McFarland standards. Then 100 μl of bacterial suspension was added to each well and incubated for 8–20 h at 28°C. To evaluate the impact of DMSO on bacterial growth, DMSO was added to the positive control group. The MIC value of each drug was defined as the lowest concentrations with no visible bacterial growth.

### 2.3 Growth Curves

Growth curves assay was carried out to determine the impact of genistein on bacterial growth. *A. hydrophila* XS-91-4-1was culture in Brain-heart infusion (BHI) medium overnight, then the bacterial culture was sub-inoculated to 100 ml fresh BHI medium at a ratio of 1:100. The culture was aliquoted into 5 flasks at a volume of 20 ml when the optical density at 600 nm (OD_600nm_) of the culture reached to 0.3. Then different concentrations of genistein were added to each flask and incubated for further 5 h at 28°C. A spectrophotometer was used to measure the OD_600nm_ values of each flask every 30 min.

### 2.4 Hemolytic Activity Assay

Hemolytic activity assay was performed as described elsewhere (Dong et al., 2017). Briefly, bacterial cultures supplemented with indicated concentrations of genistein at 28°C in a shaker incubator to obtain the OD_600nm_ to 1.5. Then bacterial supernatants were acquired by centrifugation. The hemolytic reaction system was brought up by 875 μl hemolytic buffer, 100 μl trypsin-activated supernatant and 25 μl sheep red blood cells (5 × 10^6^cells/ml). 1% Triton X-100 treated group served as positive control group. The mixtures were centrifuged to remove the unlyzed red blood cells after an incubation for 20 min at 37°C. Then the hemolytic activities of different treatment were evaluated by measuring the absorption at OD_543nm_ of the supernatants.

### 2.5 Western-Blotting Assay

Supernatants obtained from hemolytic activity assay were used for Western-blotting assay. Supernatants were boiled for 10 min after mixed with laemmli sample buffer. Samples were centrifuged and loaded to a sodium dodecyl sulfate (SDS)-polyacrylamide (12%) gel. Then proteins in the gel were transferred to a polyvinylidene fluoride membrane by a semi-dry transfer cell. The membrane was incubated with a primary anti-aerolysin polyclonal antibody after blocked with 5% non-fat milk at room temperature. Following incubated with HRP-conjugated secondary goat anti-rabbit antiserum, proteins the membrane was detected by a Supper ECL Western Blotting Substrate.

### 2.6 Biofilm Formation Assay

Biofilm formation assay was performed in 96 well plates according to previous study with some modification (Tanhay Mangoudehi et al., 2020). An overnight bacterial culture was sub-inoculated to fresh BHI medium to obtain a OD_600nm_ of 1.0. The bacterial culture was diluted with fresh medium at a ratio of 1:20 and added to each well with various concentrations of genistein. The mixtures were further incubated without shaking at 37°C for 24 h. Wells filled with fresh medium was served as negative control, while wells filled with bacterial suspension with an indicated concentration of DMSO was served as positive control. Before quantification, the values of OD_600nm_ were determined to confirm all the wells were at stationary phase. The medium and unattached bacteria were removed and then washed with PBS to remove any remaining planktonic bacterial cells. The biofilms in each well were stained with 0.5% crystal violet for 30 min after air-dried. After washing, the dye was released by addition of 30% glacial acetic acid. The quantification of the biofilm was determined by measuring the absorption at 570 nm by a microplate reader.

### 2.7 Microscopic Assay of Biofilm Formation

For microscopic assays, biofilms were grown on glass slides in a 24 well cell plate with indicated concentrations of genistein at 37°C for 24 h. The unattached bacterial cells were removed by washing with distilled water for three times and then treated as described below. For light microscopic analysis, the glass slides were air-dried and stained with 0.5% crystal violet for 3 min. After washing and drying, the biofilms on the glass slides were directly detected by a light microscope at a magnification of ×400 and captured by an inbuilt digital camera.

### 2.8 Quantitative Real-Time PCR

Bacterial cells co-cultured with indicated concentrations of genistein were acquired when OD_600nm_ of the cultures reached to 1.5. Then total RNA of the bacteria was extracted by a MolPure Bacterial RNA Kit. After removing the excessed DNA, total RNA was used to synthesize cDNA by a PrimeScript RT Master Mix. Then qRT-PCR was performed on a CFX96 Touch Real-Time PCR Detection System (Bio-Rad, California, United States). Primer pairs of detecting genes were listed in Table 1. The relative expression levels of each gene was analyzed by 2^−ΔΔCt^ method.

**TABLE 1:** Primer pairs used in qPCR assay.

Primer	Sequence (5′-3′)	PCR amplicon (bp)	References
*aerA*-F	TCT​ACC​ACC​ACC​TCC​CTG​TC	218	Dong et al. (2017)
*aerA*-R	GAC​GAA​GGT​GTG​GTT​CCA​GT		
*ahyR*-F	TTT​ACG​GGT​GAC​CTG​ATT​GAG	206	Patel et al. (2017)
*ahyR*-R	CCT​GGA​TGT​CCA​ACT​ACA​TCT​T		
*ahyI*-F	GTC​AGC​TCC​CAC​ACG​TCG​TT	202	Dong et al. (2020)
*ahyI*-R	GGG​ATG​TGG​AAT​CCC​ACC​GT		
16S rRNA-F	TAA​TAC​CGC​ATA​CGC​CCT​AC	164	Dong et al. (2017)
16S rRNA-R	ACC​GTG​TCT​CAG​TTC​CAG​TG		

### 2.9 Cell Viability Assays

The protective effect of genistein on aerolysin induced cell injury was evaluated by A549 cells according to previous study. Briefly, A549 cells obtained from ATCC was cultured in DMEM medium supplemented with 10% fetal bovine serum at 37°C with 5% CO_2_. Cells at a density of 1.5 × 10^5^ per well were seeded into a 96-well cell plate and were further incubated overnight. Then bacterial supernatants used in hemolytic assay were added to each well and co-cultured with A549 cells for further 2 h. After incubation, cell supernatants were obtained and were applied for LDH release assay using a LDH Cytotoxicity Assay Kit. Cells were washed by sterile PBS for three times and were stained by LIVE/DEAD regents. Live cells were stained with green, while dead cells were red, images were captured by a fluorescence microscope.

### 2.10 Ethics Statement

Animal studies were performed at fishery drugs clinical trials center of Yangtze River Fisheries Research Institute under the guidance of the experimental practices and standards developed by the Animal Welfare and Research Ethics Committee. The experimental protocols were approved and supervised by the animal care committee (Permit No. YFI-2020DJ-019).

### 2.11 Experimental Therapeutics

60 healthy channel catfish was divided into 3 groups, and was maintained in 100 L glass tanks for 7 days before bacterial challenge. XS-91-4-1 was cultured in BHI medium at 28°C to the mid-log phase, bacterial cells were obtained by centrifugation. After washed by sterile PBS for 3 times, the concentration of bacterial cells was adjusted to 1.5 × 10^8^ cfu/ml by a McFarland standard. Fish in positive group and genistein treated group were intraperitoneally injected with 200 μl bacterial suspension, while fish in negative group were injected with 200 μl sterile PBS. Then fish in genistein treated group were administered with 20 mg/ml genistein by a gavage needle, while fish in positive group and negative group were administered with PBS 6 h post infection and then 12- h intervals for 3 days. Deaths in each group were observed every 24 h for 8 days.

### 2.12 Statistical Analysis

Data in hemolysis, biofilm formation, relative gene expression and LDH assay were analyzed by Student’s *t*-test. The survival rate was analyzed by Kaplan-Meier estimates and log-rank test. *p* < 0.05 indicates statistical significance.

## 3 Results

### 3.1 Effect of Genistein on Bacterial Growth

The MIC of genistein against *A. hydrophila* XS-91-41 was higher than 512 μg/ml, while 4 μg/ml for enrofloxacin when determined by micro-dilution method. The results indicated that genistein had no inhibitory effect against *A. hydrophila* under our experimental conditions. Moreover, the results of growth curves assay (Figure 1B) showed that genistein had no influence to bacterial growth at concentrations ranging from 32 to 4 μg/ml in 5 h.

### 3.2 Effect of Genistein on Hemolysis of Bacterial Supernatants

**FIGURE 2:**
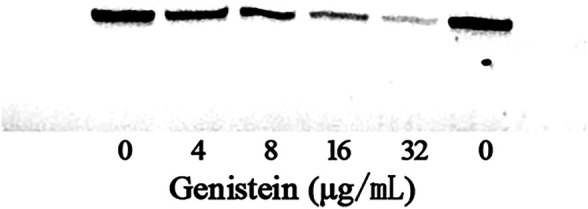
Detection of aerolysin production in bacterial supernatant with genistein by Western-blot.

The MIC and growth curves assays had demonstrated that there was no role of genistein against bacterial growth under our experimental concentrations. However, the hemolytic activity assay showed that genistein could inhibit the hemolysis induced by bacterial supernatants at a dose dependent manner following the addition of genistein in bacterial cultures. As shown in Figure 1C, the hemolysis decreased to 39.23 ± 7.46, 29.52 ± 5.55, 19.47 ± 4.60, and 15.42 ± 3.03% when co-cultured with genistein at concentrations of 4, 8, 16, 32 μg/ml, while the hemolysis of drug-free group was 74.73 ± 13.29%. The hemolytic activity of the supernatant was significantly reduced by addition of genistein at concentrations of 4 μg/ml and above. To analyze the inhibitory effect of genistein on the production of aerolysin in the supernatants, Western blot assay was performed. As desired, the levels of aerolysin in the supernatant were sequentially decreased following the increasing concentrations of genistein (Figure 2). As shown in Figure 2, there was only a little aerolysin was detected when genistein reached to 32 μg/ml. Taken together, the results revealed that genistein could reduce the hemolytic activity of bacterial supernatants when co-cultured with *A. hydrophila* at no-inhibitory concentrations by decreasing the production of aerolysin.

### 3.3 Inhibitory Effect of Genistein on Biofilm Architecture

**FIGURE 3:**
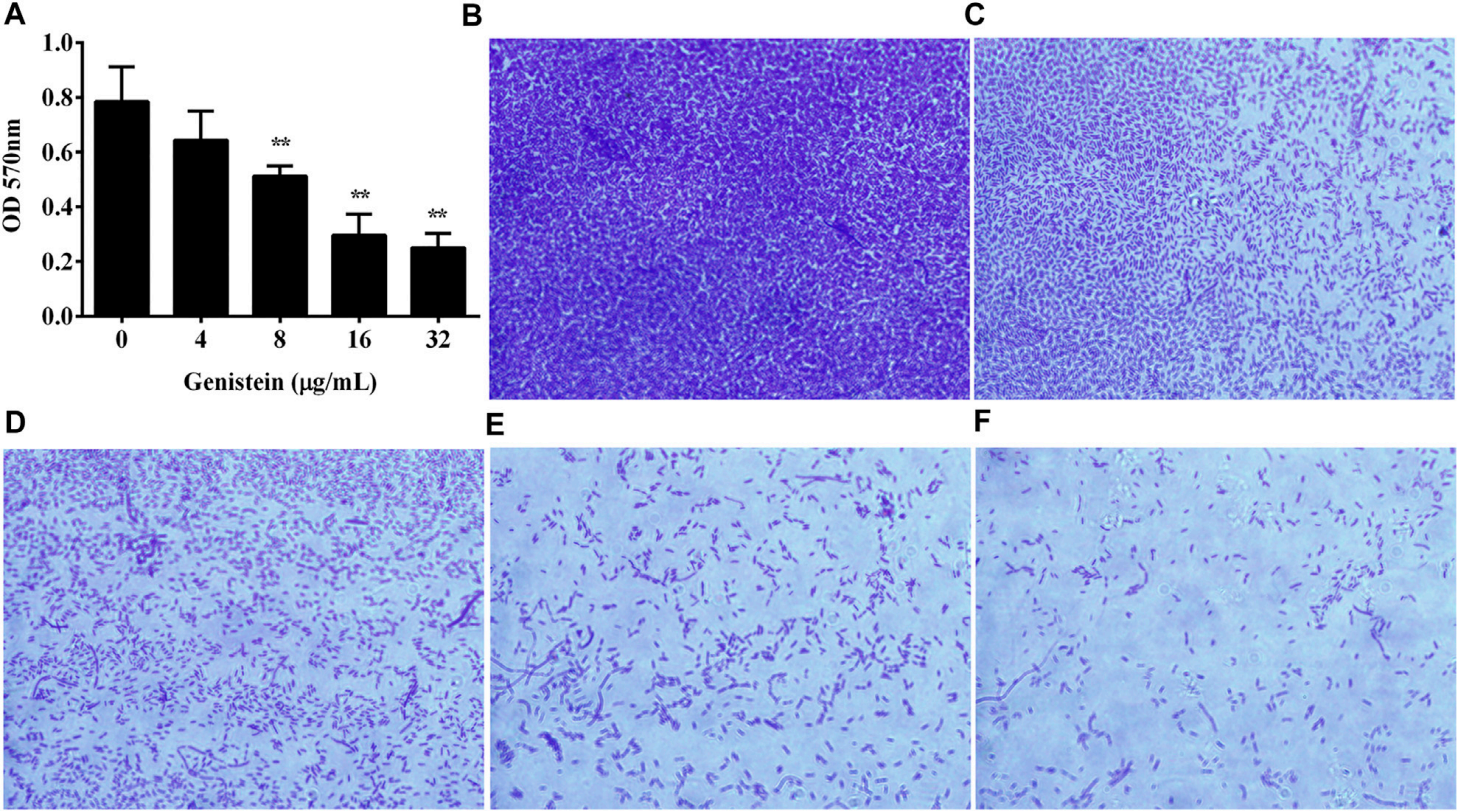
Inhibitory effects of genistein on biofilm formation. **(A)**, Determination of genistein against *A. hydrophila* biofilm formation, Data in Figure 3A represented mean value ±SD of three independent assays, *p < 0.05 and **p < 0.01 when compared with drug-free group; **(B–F)**, inhibitory effects of genistein on biofilm formation by microscopic validation; **(B)**, drug-free group; **(C)**, co-cultured with 4 μg/ml genistein; **(D)**, co-cultured with 8 μg/ml genistein; **(E)**, co-cultured with 16 μg/ml genistein; **(F)**, co-cultured with 32 μg/ml genistein.

The inhibitory effect of genistein on biofilm formation of *A. hydrophila* was determined. As shown in Figure 3A, the addition of genistein could reduce the quantity of biofilm at a dose dependent manner when determined by crystal violet staining. Biofilm formation of *A. hydrophila* was significantly inhibited when co-cultured with genistein at concentrations of 8 μg/ml and above (Figure 3A). The amount of biofilm decreased to 31.86% in group co-cultured with 32 μg/ml genistein compared with drug free group. Moreover, the inhibitory effect of genistein on biofilm formation was analyzed microscopically by testing bacterial biofilms on glass slides with indicated concentrations of genistein. As shown in Figures 3B–F, a dense biofilm was observed on the control glass slide, while a visible reduction of biofilm in the glass slide with 4 μg/ml genistein and above (Figure 3F).

### 3.4 Effect of Genistein on Transcription of Related Genes

**FIGURE 4:**
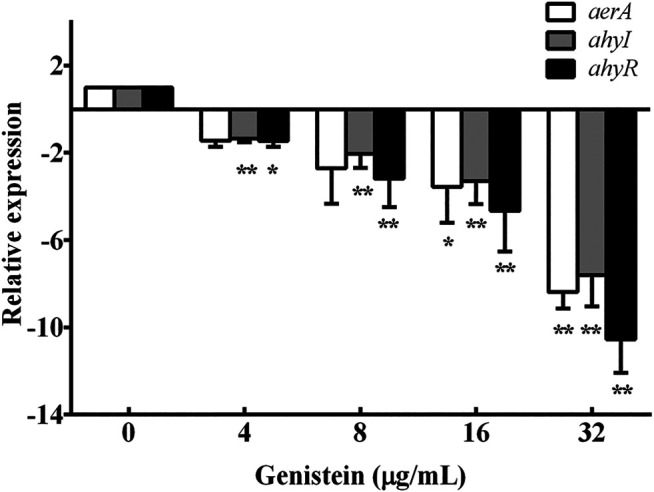
Treatment with genistein decreased the transcription levels of *aerA*, *ahyI* and *ahyR*. Transcription levels of different genes were determined by qPCR assays in triplicate, data were mean value ±SD. *p < 0.05 and **p < 0.01.

Aerolysin expression and biofilm formation of *A. hydrophila* were regulated by QS system. The results of hemolytic activity assay and biofilm formation assay indicated that genistein might be a QS inhibitor. Therefore, the transcription levels of aerolysin encoding gene *aerA* and QS regulators *ahyI* and *ahyR* were analyzed by qPCR. As shown in Figure 4, the *aerA* gene was 8.39-fold down-regulated plus 32 μg/ml genistein compared with drug-free group, while 7.61 -fold and 10.55 -fold for *ahyI* and *ahyR*, respectively (Figure 4).

### 3.5 Effect of Genistein on Aerolysin Mediated Cell Injury

**FIGURE 5:**
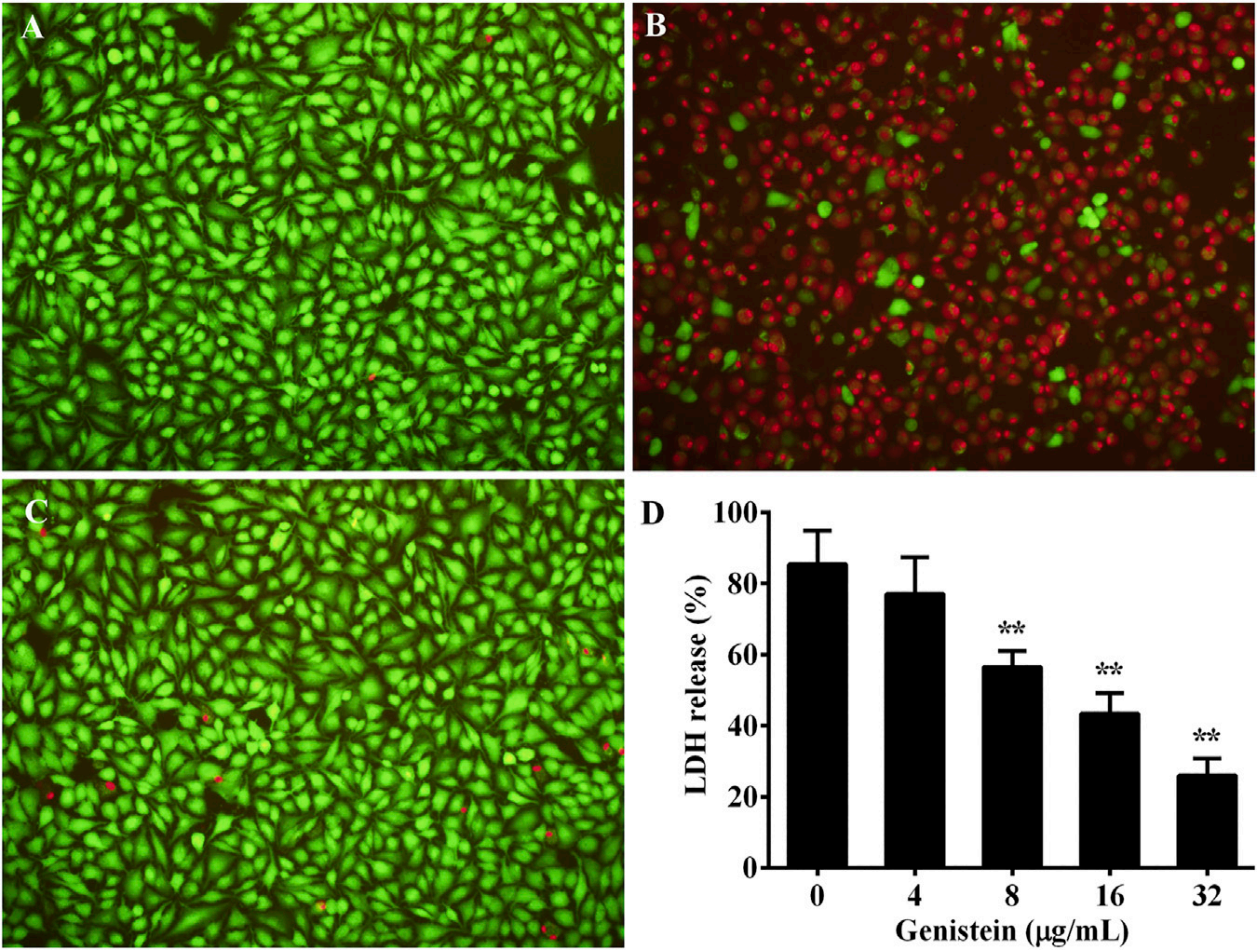
Protective effects of genistein on aerolysin mediated A549 cell injury. Cell injury induced by aerolysin was evaluated by live/dead staining assay and LDH release assay. For live/dead staining assay, live cells were stained with green, while dead were red. **(A)**, cells without any treatment; **(B)**, cells treated with drug-free supernatant; **(C)**, cells treated with supernatant obtained from bacterial culture plus 32 μg/ml genistein; **(D)**, LDH release of A549 cells treated with bacterial supernatants plus indicated concentrations of genistein, LDH assay was performed in triplicate, data were mean value ±SD. *p < 0.05 and **p < 0.01.

A549 cells was used to evaluate the protective effect of genistein on aerolysin induced cell injury. As shown in Figure 5A, cells without any treatment were stained green, indicating live cells. Dead cells staining red were observed when treated with drug-free bacterial supernatant (Figure 5B). The quantity of dead cells in genistein treated group showed a visible decrease compared with drug-free group (Figure 5C). The results indicated that genistein could reduce cell death caused by aerolysin. Moreover, cell viability was determined by LDH release assay. As shown in Figure 5D, genistein could reduce LDH release at a dose dependent manner. LDH release of A549 cells decreased to 25.94 ± 3.92% in group co-cultured with bacterial supernatant plus 32 μg/ml genistein, while 85.42 ± 7.65% of drug-free group.

### 3.6 Protective Effect of Genistein on Fish Challenged With *A. hydrophila*

**FIGURE 6:**
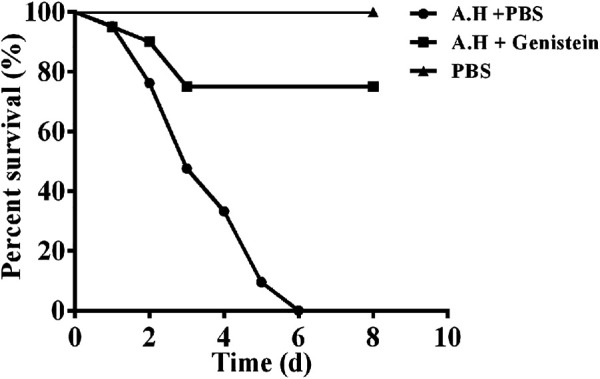
Genistein treatment improved the survival rate of channel catfish infected with *A. hydrophila*. Fish infected with *A. hydrophila* were treated with 20 mg/kg genistein or sterile PBS every 12 h for 3 days, deaths were observed for 8 days after infection. Treatment with genistein could provide a significant protection to fish challenged with *A. hydrophila* when analyzed by log-rank test (*p* < 0.0001).

*In vitro* studies have demonstrated that genistein could protect A549 cells from cell death by inhibiting the expression of aerolysin and QS system. Therefore, an *A. hydrophila* infection model of channel catfish was established to study the *in vivo* protective effect of genistein. Fish challenged with *A. hydrophila* showed heamorrhage and swelling in gill and fin. Ascites in abdominal cavity and hyperaemia in liver and kidney were found by anatomy. Moreover, body weight loss was observed in positive control group by monitoring body weight every 24 h. Deaths were occurred after 24 h post challenge. As shown in Figure 6, all fish in positive control group were dead at 6 days post infection, while fish in genistein treated group showed 75% survive which was statistically significant (*p* < 0.01) compared with positive control. No death was observed in negative control group during all the experimental period. The results demonstrated that treatment with genistein could significant decrease the pathogenesis of *A. hydrophila* in a channel catfish infection model.

## 4 Discussion

The growth of human population and development of agriculture has resulted in the emergence of infectious diseases (McMichael, 2004). The introduction of penicillin and other antibiotics since 1940s has significantly increased the survival rate of human suffered from bacterial infections (Coates et al., 2002). However, antibiotic resistance observed in a few years after the deployment of antibiotics, which had become one of the biggest challenges of the 21th century (Palumbi, 2001; Rasko and Sperandio, 2010). Antibiotics were widely used in aquaculture in attempts to dealing with bacterial infections. The wide and frequent use of antibiotics in fish farming has led to the development of antibiotic resistance in aquatic bacterial pathogens (Defoirdt et al., 2011). In some cases, cure failure was observed when treated bacterial infections by antibiotics. *A. hydrophila*, one of the most common bacterial pathogens in aquaculture environments, has been reported to be resistant to a number of antibiotics such as ciprofloxacin, tetracycline and rifampin (Hossain et al., 2018). Resistant genes of *A. hydrophila* was located on transferable plasmids and integrons which could transmit to animal and human bacterial pathogens (Defoirdt et al., 2011). Antibiotic resistant bacteria have become an important public health threat. Therefore, alternative strategies were needed to overcome microbial infections. In recent year, targeting virulence has become a novel approach for antimicrobial therapy with the increasing knowledge of bacterial pathogenesis (Clatworthy et al., 2007). Virulence factors of pathogenic bacteria are required to cause host damage and diseases. Thus, anti-virulence strategy was to decrease the disruption caused by virulence factors to the host. Then the attenuated bacterial strain will be cleared by the host immune response (Clatworthy et al., 2007). Toxins, adhesions, secretion systems and regulations of virulence can be selected as targets identifying alternative drugs combating bacterial infections. The pathogenesis of *A. hydrophila* was composed by toxins, structural components, secretion systems, biofilm and proteins associated with metals (Fernandez-Bravo and Figueras, 2020). Aerolysin can form a channel pore on target cells by binding to the glycophospha-tidylinositol (GPI) anchored proteins and results in cell death (Singh et al., 2008). Biofilms can increase the survival rate and contamination of bacterial pathogens in aquatic environment (Packiavathy et al., 2013), which is critical for bacterial pathogens in establishing infections and developing diseases (Tanhay Mangoudehi et al., 2020). Thus, aerolysin, the chief virulence factor, and QS, virulence regulating system, were selected as targets in drug discovery.

Herbal medicines have a long history in the treatments of human and animal diseases. Moreover, herbal medicines are the only resources for treating infectious diseases in some developing countries (Savoia, 2012). Natural compounds extracted from herbal medicines have been proven to play critical roles in the anti-infective area (Newman and Cragg, 2012). Therefore, herbal medicines have attracted the attention of researchers in developing novel drugs against bacterial infections. Bacterial infections in fish farming are one of the major concerns limiting the healthy development of aquaculture. Although the introduction of antibiotics to aquaculture decreases mortality and avoid huge economic losses in aquaculture, the therapy is limited because of the emergence of antibiotic resistance, adverse effects to aquatic environments and human health (Valladao et al., 2015). Therefore, herbal medicines with anti-bacterial, anti-inflammatory, anti-oxidative and anti-parasitic activities have been widely used in aquaculture as an alternative for treating fish diseases (Valladao et al., 2015; Perez-Sanchez et al., 2018).

In the present study, natural compounds were used to screen drugs inhibiting the pathogenesis of *A. hydrophila*. As expected, genistein was found to provide a significant protection to channel catfish challenged with *A. hydrophila*. Genistein is well-known as a tyrosine-specific-protein kinase that can prevent a number of microorganisms into target cells (Vela et al., 2008; Siriviriyakul et al., 2020). In aquaculture, genistein has been recognized as an additive to improve body function. Fickler et al. (2019) evaluated the ability of genistein on biosynthesis of omega-3 fatty acids eicosapentaenoic acid and docosahexaenoic acid in *Oncorhynchus mykiss*, the results showed that the addition of genistein could stimulate the biosynthesis of docosahexaenoic acid at a moderate extent (Fickler et al., 2019). Moreover, Jourdehi et al. (2014) demonstrated that genistein could be used as an additive to diets for inducing ovary development of *Huso* (Jourdehi et al., 2014). Tan et al. (1998) demonstrated that genistein could be a tyrosine kinase inhibitor that postpone the internalization of *A. hydrophila* into host cells (Tan et al., 1998). These reports indicated that genistein could be a useful agent in aquaculture. However, there was little study of genistein against aquatic bacteria. Verdrengh et al. (2004) found that genistein could inhibit the growth of *Staphylococcus aureus* (*S. aureus*) including methicillin-resistant strains of *S. aureus in vitro* by disturbing the stabilization of the covalent topoisomerase II-DNA cleavage complex, indicating that genistein could be a potent anti-staphylococcal agent (Verdrengh et al., 2004). Hong et al. (2006) reported that genistein had antibacterial activity to opportunistic bacterial pathogens, such as *Bacillus anthracis* and *S. aureus* at 100 μM (Hong et al., 2006). Here we found that the MIC of genistein against *A. hydrophila* XS-91-4-1 was higher than 512 μg/ml. Taken the results of growth curves together, these indicated that genistein could hardly inhibit the growth of *A. hydrophila* under our experimental conditions.

Although the antibacterial activity of genistein to different kinds of bacterial organisms was different, it has been reported as virulence inhibitors against bacterial pathogens (Oh et al., 2010; Kim and Lee, 2020; Gautam et al., 2021). Kim and Lee (2020) found that genistein-induced nitric oxide could cause apoptosis-like death in *Escherichia coli* (Kim and Lee, 2020). Gautam et al. (2021) investigated the inhibitory effect of genistein against *Acinetobacter baumannii* (*A. baumannii*) by disrupting virulence (Gautam et al., 2021). Inhibition of biofilm, surface motility and increasing expression of polyP degrading enzyme were observed, indicating that genistein could be a potent anti- *A. baumannii* agent by inhibiting virulence. In the present study, genistein could affect the production of aerolysin in the bacterial supernatants and biofilm formation at a dose-dependent manner. Mutagenesis of *ahyI* and *ahyR* genes could reduce the production of exoprotease by abolishing the ability of producing QS signal N-butanoyl-L-homoserine lactone in *A. hydrophila* (Swift et al., 1999). QS system of pathogenic *A. hydrophila* involved in the regulation of biofilm formation, hemolytic activity and motility and other phenotypes (Rama Devi et al., 2016). Therefore, the transcription of aerolysin encoding gene *aerA* and QS related gene *ahyI* and *ahyR* were detected according to previous study (Patel et al., 2017). All three detected genes were down-regulated after treatment of genistein. The findings in qPCR assay indicated that genistein could inhibit the production of aerolysin and biofilm formation of *A. hydrophila* by inhibiting QS system. Moreover, previous studies demonstrated that drugs inhibiting aerolysin production and biofilm via inhibiting QS could decrease the pathogenesis of *A. hydrophila* in animal models (Rama Devi et al., 2016; Patel et al., 2017; Dong et al., 2020). Thus, it is reasonable to believe that genistein can protect fish infected with *A. hydrophila*. As desired, a significantly protective effect was achieved by administering with 20 mg/kg genistein to fish infected with *A. hydrophila*. Genistein could inhibit the pathogenesis of *A. hydrophila* XS-91-4-1 strain both *in vitro* and *in vivo* under our experimental conditions, but the expression levels of aerolysin in different sources of *A. hydrophila* was distinctive. Therefore, the dosage of genistein should be adjusted according to the pathogenesis of *A. hydrophila* if used for treatment of *A. hydrophila* associated infections. Taken together, genistein can be developed as a novel fishery drug by inhibiting the pathogenesis of *A. hydrophila* rather than affecting the growth of the bacterium. Genistein can be an alternative approach against *A. hydrophila* infections which might decrease the use of antibiotics and delay the emergence of antibiotic resistance in aquaculture. Moreover, the findings provide a novel approach identifying drugs against bacterial infections in aquaculture.

## 5 Conclusion

The present study investigated the pharmacological activity of genistein against the pathogenesis of *A. hydrophila* by inhibiting QS system. The results found that genistein could decrease the production of aerolysin and biofilm at concentrations without anti-*A. hydrophila* activity by inhibiting QS mechanism. Moreover, genistein could provide a protection both *in vitro* and *in vivo* against *A. hydrophila*. These findings indicated that genistein can be a promising candidate in the treatment of *A. hydrophila* infections.

## Data Availability

The raw data supporting the conclusion of this article will be made available by the authors, without undue reservation.
